# Improving Bond Performance and Reducing Cross-Linker Dosage of Soy Protein Adhesive via Hyper-Branched and Organic–Inorganic Hybrid Structures

**DOI:** 10.3390/nano13010203

**Published:** 2023-01-02

**Authors:** Zheng Cui, Yecheng Xu, Gang Sun, Lai Peng, Jianzhang Li, Jing Luo, Qiang Gao

**Affiliations:** 1 MOE Key Laboratory of Wooden Material Science and Application & Beijing Key Laboratory of Wood Science and Engineering, Beijing Forestry University, Beijing 100083, China; 2 College of Materials Science and Engineering, Nanjing Forestry University, Longpan Road 159, Xuanwu District, Nanjing 210037, China; 3 Arte Mundi Group Co., Ltd., Shanghai 201700, China

**Keywords:** bonding performance, soy protein adhesive, organic–inorganic hybrid, hyper-branched structure, cross-linker dosage

## Abstract

Eco-friendly soybean protein adhesives could be an ideal substitute for replacing traditional formaldehyde-based adhesives in wood industry. However, a large number of cross-linking agents are required in soy protein adhesive formulations to obtain sufficiently performing properties. Inspired by the high performance of nacre and branched structures, a hyper-branched amine (HBPA) was synthesized and grafted to graphene oxide (GO), generating a hyper-branched amine-functionalized GO (FGO). A novel soy protein-based adhesive was developed by mixing FGO with soy protein (SPI) and a low dose polyamidoamine-epichlorohydrin (PAE). Results showed that the addition of only 0.4 wt% FGO and 0.75 wt% PAE to the SPI adhesive formulation enhanced the wet shear strength of plywood to 1.18 MPa, which was 181% higher than that of the adhesive without enhancement. The enhanced performance is attributed to the denser cross-linking structure and improved toughness of the adhesive layer. Using FGO in the adhesive formulation also greatly reduced the concentration of the additive cross-linker by up to 78.6% when compared with values reported in the literature. Thus, using a hyper-branched functionalized nano-material to form an organic–inorganic hybrid structure is an effective and efficient strategy to reinforce the composites and polymers. It significantly reduces the chemical additive levels, and is a practical way to develop a sustainable product.

## 1. Introduction

The development of biomass adhesives to replace carcinogenic formaldehyde adhesives is a significant advancement [1,2,3]. Vegetable protein adhesives, especially soy protein-based adhesives (SPIAs) have attracted significant attention from the scientific community because of their richness, degradability, and lack of toxicity as harmless substitutes for formaldehyde-based adhesives [4,5,6]. However, the poor water resistance and inadequate mechanical properties of SPIAs limit the practical application in wood panel fabrication industry [7,8,9]. Researchers have used a variety of chemical methods to improve their bond performance, including surfactants [10], graft-modification [11], etc. Enzyme-modification has also been proved to be a feasible method. Soybean protein was degraded into soybean protein polypeptide by bromelain hydrolysis, and then flexible epoxy crosslinking agent, tannic acid, and Zn2+ were introduced to react with the active group of soybean protein polypeptide. By constructing a triple network structure including covalent bond, hydrogen bond, and ionic bond, the viscosity of soy protein adhesive was reduced, and the strength, toughness, and anti-mildew properties were improved [12]. Different types of natural tannins have also been selected to modify soybean protein adhesives to prepare plywood. It has been proved that under the action of heat, the dominant covalent bond can be formed between tannin components and amino acids [13,14]. Among the chemical methods, cross-linking is preferred by most researchers [15]. Effective cross-linking agents include triglycidylamine [16], polyamidoamine-epichlorohydrin [17,18], and other epoxy cross-linking agents. These modified adhesives have been successfully used to fabricate high water-resistant plywood and blockboard in the wood panel industry [19,20]. However, in order to ensure adequate and stable bonding of the resultant adhesive, a large dosage of hazardous and energy-intensive chemical agents, especially denaturant and cross-linking agents, is required for adhesive formulation [21]. In addition, the high dosage of cross-linker leads to brittleness of the cured adhesive, which limits its bond performance [22]. Therefore, obtaining safe adhesives and wood panel products based on novel SPIAs with improved adhesion and toughness and reduced amounts of cross-linker is a challenge.

The combination of inorganic nano-fillers such as aragonite flakes and organic matter such as proteins layered into a “brick-and-mortar” structure contributes to the high strength and toughness of the nacre [23,24,25]. Composites with high strength and toughness have been developed, inspired by this organic–inorganic hybrid structure [26,27]. Graphene oxide (GO) is often selected as an inorganic nano-filler due to its superior Young’s modulus, excellent thermal stability, high mechanical strength and flexibility, and a variety of other beneficial properties [28,29,30], which theoretically improve the overall performance of SPIAs. However, GO is not easy to use to reinforce wood adhesives due to its large surface area, high surface energy, and strong van der Waals interactions, which lead to poor dispersion and easy aggregation in the adhesive system [31,32]. Further, due to the weak interaction between the GO and the adhesive matrix, once the stress increases to a certain threshold, interface slippage limits the mechanical properties. Therefore, these problems must be solved to obtain an optimal bonding performance of the adhesive with GO.

Several branching structures exist in nature, such as leaf, lighting, and tree root, which contribute to their high performance. Inspired by the branched structure of materials, hyper-branched polymers exhibit unique properties compared with commonly used linear polymers, such as high reactivity, good shear resistance, and large internal voids [33,34]. Their highly branched three-dimensional structure and the large number of functional groups are a research hotspot [35]. Therefore, the use of hyperbranched polymers may resolve these limitations of GO. The formation of hyperbranched cross-linking structure improves the adhesive performance and reduces the cross-linker dosage.

**Scheme 1:**
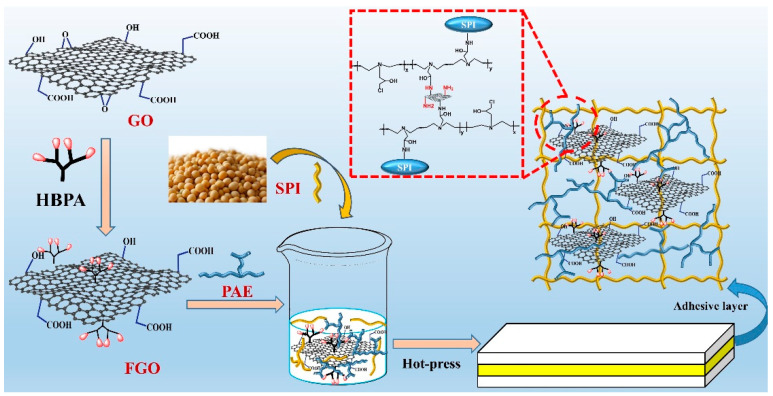
FGO preparation and cross-linking mechanism within SPI-based adhesives.

In our strategy, a hyperbranched polyamide was synthesized and grafted onto GO via reaction between amino and carboxide/epoxy groups to develop a hyper-branched amine-functionalized GO (FGO). This process improves the dispersion of GO. The amino groups on GO form covalent bonds to reinforce the interface between GO and adhesive matrix. In order to clearly analyze the effect of FGO on the performance of SPIA, the soy protein was mixed with FGO and a small amount of cross-linker (Scheme 1). The functional groups, residual rate, toughness, fracture surface morphology, and bonding performance of the resultant SPIAs were measured. The reinforcement mechanism was discussed and the cross-linker dosage in the adhesive formulation was compared with that reported in the literature. This strategy can be used to analyze commercially available SPIAs. Of equal importance, this strategy also can be extended to improve the dispersibility of other nano materials used to reinforce the performance of composite materials, such as films, hydrogels, and polymers.

## 2. Materials and Methods

### 2.1. Materials

GO (1–2 layers) with an average diameter of 10 µm and a thickness of 1.5 nm was obtained from Suzhou Tanfeng Graphene Technology Co., Ltd. (Jiangsu, China). Soy protein isolate (SPI, 96% protein content) was supplied by Yuwang Ecological Food Industry Co., Ltd. (Shandong, China). Poplar veneer (400 × 400 × 1.5 mm, 8% moisture content) was acquired from Arte Mundi Aesthetic Home Furnishings Co., Ltd. (Shanghai, China). All other chemical reagents were provided by Beijing Chemical Reagents Co., Ltd. (Beijing, China).

### 2.2. Preparation of the Hyper-Branched Amine (HBPA) and Polyamidoamine-Epichlorohydrin (PAE)

**Scheme 2:**
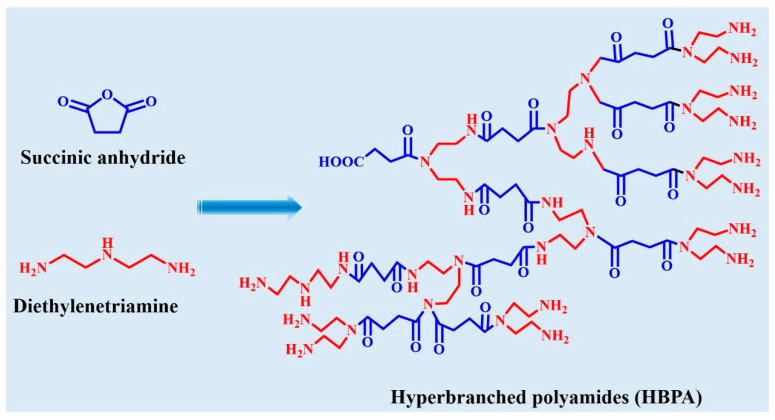
Chemical reaction synthesis of HBPA.

Hyper-branched amine (HBPA) was manufactured by adding succinic anhydride and diethylenetriamine to four flasks equipped with thermometers, condensation tubes, and agitators in a 0.8:1 molar ratio. The temperature of the mixture was increased to 140 °C, and the reaction was conducted for another 3 h. Finally, HBPA was obtained by cooling the mixture to room temperature. The reaction procedure is presented in Scheme 2.

**Scheme 3:**
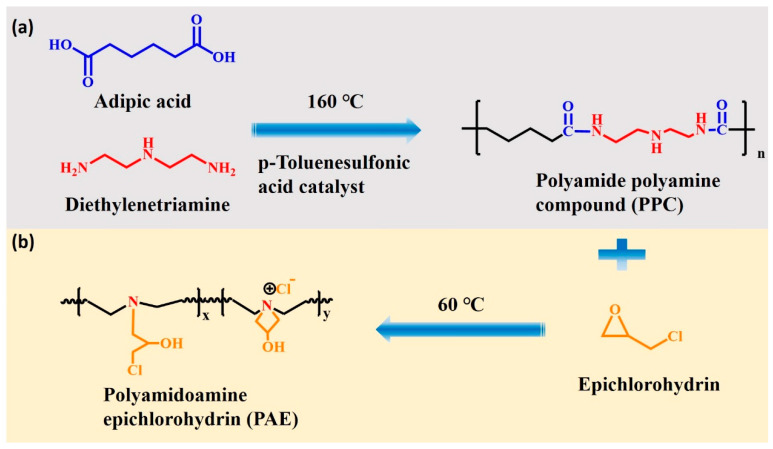
(**a**) Chemical reaction synthesis of prepolymer (PPC); (**b**) Chemical reaction synthesis of PAE.

The preparation method of polyamidoamine-epichlorohydrin (PAE) was as follows: 29.7 g diethylenetriamine and 1wt% H_2_SO_4_ were placed in a three-necked flask and heated to 130 °C while being simultaneously stirred. Next, 40 g adipic acid was added to the mixture, and the temperature was raised to 165 °C. The mixture was then subjected to a dehydration polycondensation reaction in the molten state for 3 h. The solid content was regulated by adding distilled water to obtain 50% prepolymer (PPC). Then, 25 g of the result was added in a solution form to a clean, three-necked flask, followed by dropwise addition of 18g epichlorohydrin under stirring. The solution was then allowed to condense for 1 h at 60 °C. H_2_SO_4_ was used to reduce the pH to 6, and the water content was adjusted to 12.5% [36]. The reaction procedure is presented in Scheme 3.

### 2.3. Preparation of FGO

**Scheme 4:**
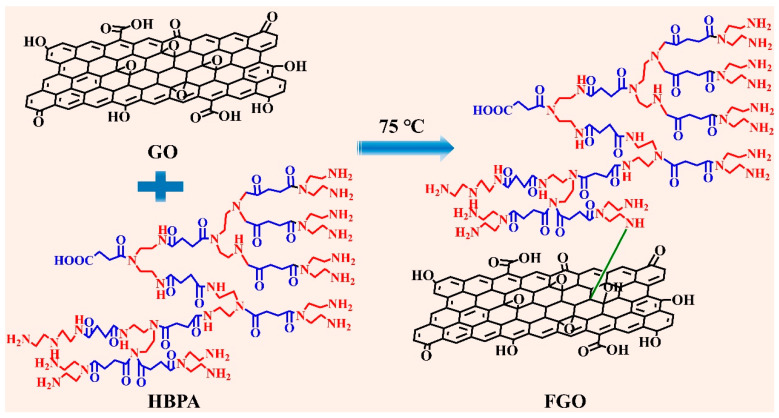
Chemical synthesis of FGO.

FGO was synthesized with the epoxy group ring-opening reaction between GO and HBPA. An amount of 1g GO was used to manufacture a 4 g/L solution. To begin, the GO was dispersed in deionized water for 1 h using a cell disrupter, after which 200 mL 20 g/L HBPA was added dropwise to the GO solvent. Next, the mixture was left to react at 75 ° C for 1 h, then cooled to normal atmospheric temperature. The sample was then centrifuged at 8000 rpm for 5 min, and washed 5 times with deionized water to remove unreacted HBPA. The reaction procedure is presented in Scheme 4.

### 2.4. Preparation of Adhesives

Pure SPIA, i.e., the control, was prepared by mechanically stirring a mixture comprising of 8 g SPI and 92 g deionized water for 10 min at room temperature. The SPI/PAE adhesive was prepared by stirring a solution of 8 g SPI in 86 g deionized water for 10 min, followed by the addition of 6 g PAE. To prepare the FGO-modified SPIA, FGO was first ultrasonically dispersed in deionized water for 1 h. SPI was then added twice, in 4 g increments. After mixing, the sample was ultrasonically cleaned for 10 min to facilitate uniform dispersion of FGO in the SPIA system. The adhesive formulations are presented in Table 1.

### 2.5. Characterization of FGO and Adhesive Samples

#### 2.5.1. Attenuated Total Reflectance (ATR) Spectroscopy 

Each adhesive sample was solidified at 120 ± 2 °C for 3 h to obtain a constant weight, and then ground to 200 mesh in a ceramic mortar. HPBA and FGO powders were dried at 100 °C, ground to 200 mesh, and GO powders were not treated. The ATR spectra of the powders were collected from 600 to 4000 cm^−1^ using a Nicolet 6700 spectrophotometer (Thermo Scientific, Pittsburgh, PA, USA), using an ATR accessory with a diamond ATR crystal. 

#### 2.5.2. Thermogravimetric (TG) Measurement

The water dispersion loss of water-based adhesive in the thermal process of TGA analysis causes inaccurate determination information, so the pre-cured adhesive was selected to indirectly measure the structure difference among the different adhesives. Each adhesive sample was solidified at 120 ± 2 °C for 3 h to obtain an invariant weight, then ground into a powder. The thermal stability of the cured adhesives was tested via TG analysis using TA Q50 (Waters, New Castle, DE, USA).

#### 2.5.3. X-ray Photoelectron Spectroscopy (XPS)

XPS (Axis Ultra DLD Kratos AXIS SUPRA) was used to analyze the elemental composition of GO and FGO via monochromatic Al K-alpha radiation with energy and resolution parameters of 50 eV and 0.1 eV, respectively.

#### 2.5.4. Crack Observation

Similar amounts of adhesive samples were evenly applied on glass slides, which were subsequently transferred to an oven at 120 ± 2 ℃ for 2 h. A digital camera (EOS M50 Mark II, Canon, Japan) was used to photograph the shape of the cured adhesive layer.

#### 2.5.5. Boiling Water Resistance and Swelling Rate 

To begin, a 40–60 mesh adhesive sample was placed at 25 °C and 70% relative humidity for 24 h [37]. The volume (V_1_) of a 1.5 g (m_1_) sample was measured using a graduated cylinder, then mixed with 150 g deionized water. The solution was then boiled and the sample was left to react with the boiling water for 3 h. Next, the sample was filtered and the volume (V_2_) of the remaining sample was measured. The residue was then dried to a constant weight (m_2_). The boiling water resistance (BWR) and swelling ratios were determined using Equations (1) and (2) as follows.

Boiling water resistance (BWR%) = m_2_/m_1_ × 100% (1)

Swelling ratio = (V_2_ − V_1_)/V_1_ × 100% (2)

#### 2.5.6. Morphological Analysis of Adhesive Layer Failure Surface

In the shear test, the wood failure rate is the percentage of the area of the wood area remaining on the damaged surface and the shear area of the specimen, which determines the adhesive quality. The macro-surface morphology of the damaged plywood surface was analyzed using a stereomicroscope (XS-18, Jiangnan Optoelectronics (Group) Co., Ltd., China).

### 2.6. Preparation of Plywood Samples

**Figure 1:**
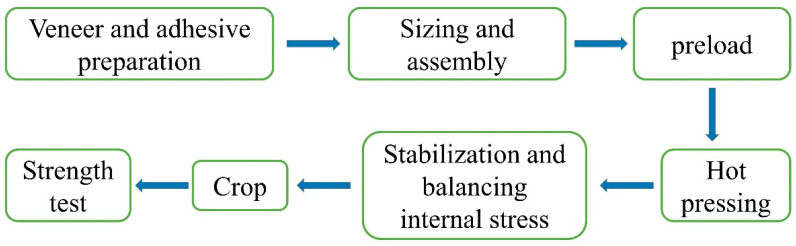
Flow chart of plywood production.

Three-ply plywood was prepared based on the procedure in outlined Figure 1 with 180 g/m^2^ of adhesive spread on the veneer surface. Next, the veneer texture was stacked vertically and pressed at 120 °C and 1 MPa for 315 s under hot press [38].

### 2.7. Bonding Strength

The wet shear strength was tested in accordance with the China National Standard GB/T17657-2013. After hot pressing, the three-ply plywood was transferred into a room at 25 °C for 24 h. Plywood samples with dimensions of 100 mm × 25 mm (glue area of 25 ± 25 mm) were cut from the plywood panels. The cut test piece was immersed in water at 63 ± 2 °C for 3 h and then cooled at room temperature for 10 min before conducting the tension test.

## 3. Results and Discussion

### 3.1. Characterization of FGO

**Figure 2:**
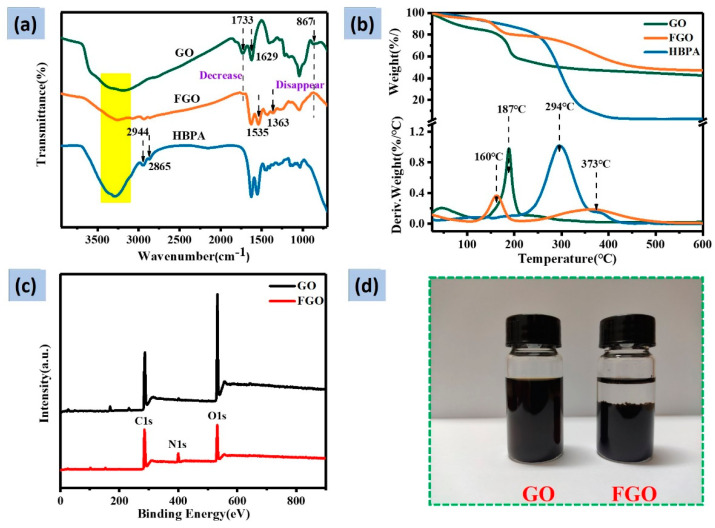
(**a**) FTIR spectra of GO, FGO, and HBPA; (**b**) TG and DTG curves of GO, FGO and HBPA; (**c**) XPS of GO and FGO; (**d**) Aqueous Solutions of GO and FGO.

Figure 2a shows the Fourier transform infrared (FTIR) spectra of the prepared HBPA compound, GO, and FGO. The absorbance peak of GO in the yellow area is attributed to the hydroxyl O-H and the peak at 1733 cm^−1^ is assigned to the carboxyl C=O. The aromatic group peak C=C appears at 1629 cm^−1^. The absorption peak at 867 cm^−1^ represents the characteristic epoxy group. These GO characterization peaks are in accordance with the results of Shi’s study [39]. In the FGO spectrum, the -CH_2_- group stretching vibration peaks are observed at 2944 cm^−1^ and 2865 cm^−1^, while the secondary amide N-H bending and C-N stretching peaks appear at 1535 cm^−1^ and 1363 cm^−1^, respectively. However, the loss of characteristic epoxy group at 867 cm^−1^ indicates that the GO epoxy group reacted with the N-H in HBPA via a ring-opening reaction [40]. A large decrease is observed in the carboxy peak intensity at 1733 cm^−1^ in the FGO spectrum, which is attributed to the GO reduction by HBPA [41]. The addition of HBPA significantly decreased the wide absorption peak of -OH in the FGO spectrum, which may be attributed to the reaction between the grafted amino group and the hydroxyl group. The above results demonstrate the successful preparation of amino-functionalized GO.

Figure 2b shows the TGA results of GO, FGO, and HBPA. For the GO, the mass loss below 100 °C is attributed to the evaporation of residual moisture in the sample. The maximum mass degradation rate of GO occurs at 187 °C and 42.13% of weight loss is observed between 100 °C and 500 °C, which is attributed to the loss of oxygen-containing groups on the GO surface [42]. The grafting of HBPA yielded two distinct degradation peaks of FGO at 160 °C and 373 °C. The weight loss of FGO from 100 °C to 200 °C corresponds to the unreacted oxygen-containing functional groups on the GO surface, while the weight loss from 200 °C to 500 °C is due to the degradation of the hyper-branched functional compound. Compared with the GO curve, the first FGO peak was reduced from 187 °C to 160 °C. Compared with the HBPA curve, the second FGO peak increased from 294 °C to 373 °C, indicating the formation of a connected structure instead of just blending between GO and HBPA. These results also demonstrate that HBPA was successfully grafted onto the GO surface.

XPS was used to observe changes in surface element chemistry before and after GO functionalization [43]. As shown in Figure 2c, a new N1s peak occurred in the FGO’s spectra due to the introduction of the amino group of HBPA. At the same time, the C/N/O atomic concentration ratio in GO was 68.2/1.17/30.62, which was altered to 72.78/7.72/19.50 in FGO. The decreasing O and increasing N content indicate that HBPA was successfully introduced onto the GO surface.

Figure 2d shows a 1 mg/mL GO and FGO aqueous solution prepared with a cell disrupter for 1 h. It was allowed to stand for one week. The GO has good dispersibility in water. After amino functionalization by HBPA, the number of hydrogen bonds between the FGOs were increased, resulting in stratification in water [44]. The results further support the successful synthesis of FGO.

### 3.2. Performance Analysis of Adhesives

#### 3.2.1. Effects of FGO on the Thermal Properties of SPI-Based Composites

**Figure 3:**
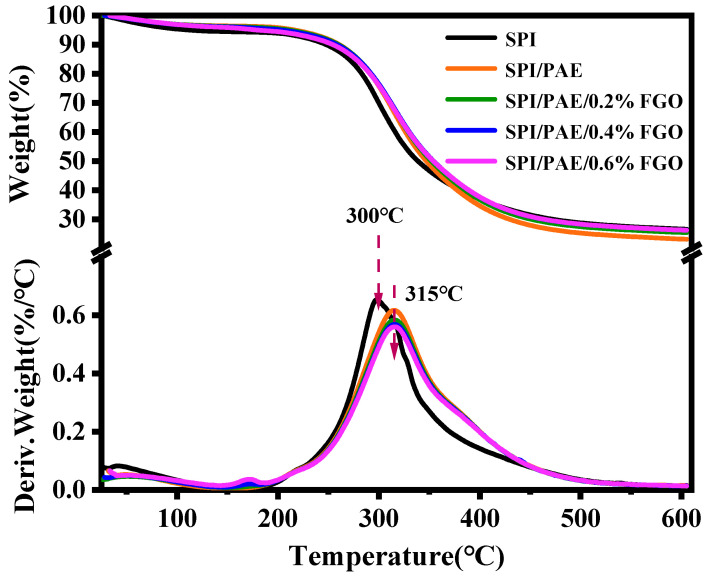
Thermal degradation curves of cured adhesives.

The TG and DTG curves of different adhesives are shown in Figure 3. The maximum SPI degradation rate corresponds to a temperature of 300 °C, and is attributed to backbone peptide bond decomposition in the soy protein [45]. With the introduction of PAE, this peak temperature increased to 315 °C, which is due to the formation of cross-linked structure in the adhesive. The degradation peak intensity gradually decreased with the introduction of FGO. These results indicate that the incorporation of FGO into the cross-linking structure yielded a differently cross-linked structure, which improved the thermal stability of the adhesive.

The temperatures at 5%, 10%, and 50% of mass loss of different adhesives are shown in Table 2. The temperatures of SPIA with mass losses of 5%, 10%, and 50% were 111.8 °C, 247.8 °C, and 338.9 °C, respectively. When PAE was added to the adhesive, the above temperatures increased significantly to 211.8 °C, 255.8 °C and 342.3 °C, respectively, indicating the formation of a dense cross-linked structure. When FGO was added to the adhesive, the above temperatures increased first and then decreased, which may be attributed to the cross-linking structural changes induced by excessive levels of hyperbranched materials.

#### 3.2.2. Boiling Water Resistance of Cured Adhesives

**Figure 4:**
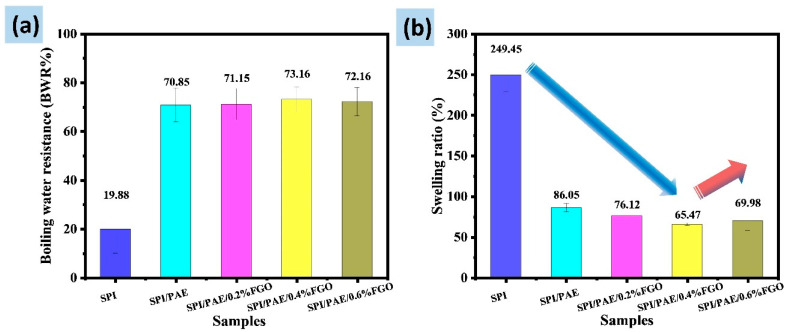
(**a**) Boiling water resistance and (**b**) swelling ratios of different adhesives.

The sol-gel method was used to evaluate the water resistance of the adhesives, as shown in Figure 4. SPI adhesives exhibit low water resistance because the hydrogen bond between –OH and/or -NH_2_ on the protein molecular chain and the carbonyl group on the peptide bond and/or the carboxylic acid contributed to the bonding strength primarily [46]. As shown in Figure 4a, after the sample was treated in a boiling water bath, the boiling water resistance (BWR%) of pure SPI sample was 19.88%, indicating that the SPIA has low water resistance. When the PAE was introduced, the BWR% of the SPI/PAE adhesive increased to 70.85%, because the N-hetero butyl group on the PAE reacted with the SPI carboxyl and amino groups to form a cross-linking structure, which greatly improved the water resistance. When FGO was added, the boiling water resistance was further increased. For example, the value of SPI/PAE/FGO-0.4 increased to 73.16%, which is attributed to the reaction between FGO and PAE to form a densely cross-linked structure in the adhesive, thereby further improving its water resistance. Interestingly, when the concentration of additive FGO was raised to 0.6 g, the BWR% of the adhesive decreased due to the introduction of excessive amino groups. The water resistance was also confirmed by the swelling value, which showed similar results (Figure 4b). The swelling ratio of pure SPI sample was 249.45%, indicating that the SPIA had low water resistance. The swelling ratio greatly decreased to 86.05% with the addition of PAE. The addition of 0.4 g FGO further reduced the swelling ratio to 65.47%. However, the value of SPI/PAE/FGO-0.6 increased to 69.98% by the introduction of excessive amino groups.

#### 3.2.3. Adhesive Properties and Mechanisms in Plywood Applications

**Figure 5:**
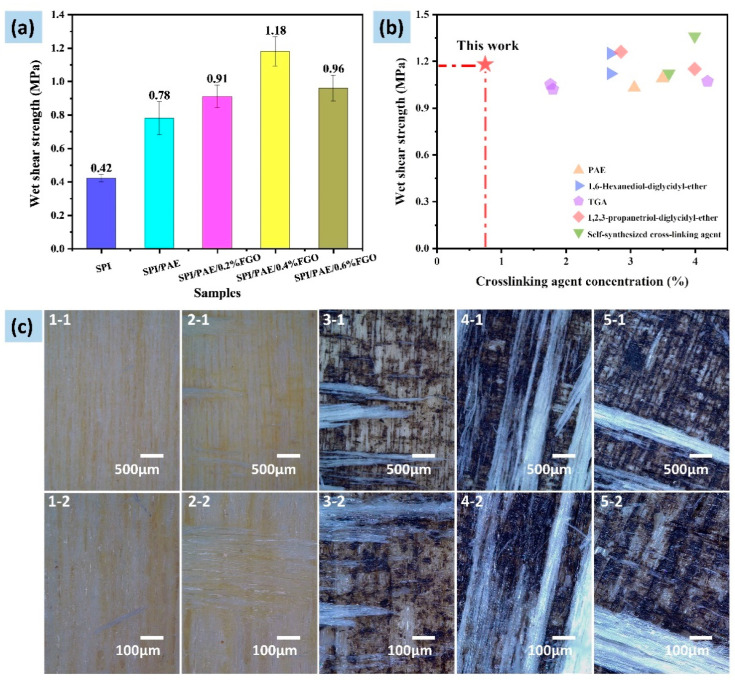
(**a**) Wet shear strength of different adhesives; (**b**) Comparison of the wet shear strengths and cross-linking agent concentrations between SPI/PAE/0.4%FGO and the other adhesives [15,20,46,47,48,49,50,51,52,53,54]; (**c**) Macro-surface morphology of the damaged plywood surface: 1-1,1-2: SPI adhesives, 2-1,2-2: SPI/PAE adhesives, 3-1,3-2: SPI/PAE/0.2% FGO adhesives, 4-1,4-2: SPI/PAE/0.4% FGO adhesives, 5-1,5-2: SPI/PAE/0.6% FGO adhesives.

**Figure 6:**
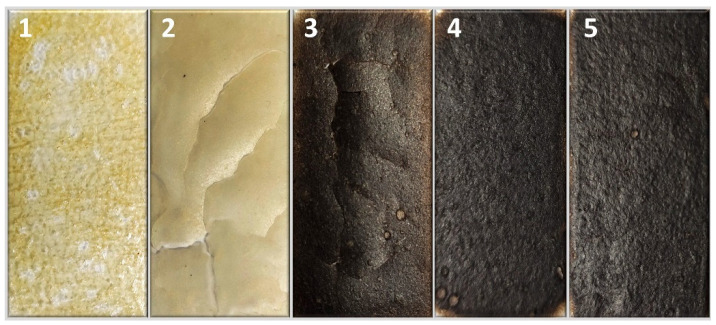
Adhesive cracks 1: SPI adhesives, 2: SPI/PAE adhesives, 3: SPI/PAE/0.2% FGO adhesives, 4: SPI/PAE/0.4% FGO adhesives, and 5: SPI/PAE/0.6% FGO adhesives.

A good adhesive tends to force the sample to break in the wooden substrate. The wet shear strength of plywood is presented in Figure 5a. Figure 5c shows the macro-surface morphology of wood treated with different adhesives tested for bonding strength. The wet shear strength of SPIA was 0.42 MPa, far below the Chinese national standard (≥0.7 MPa). The failure of the plywood prepared with the SPIA exhibits a smooth and clean surface, with almost no sign of damage. The wood failure rate of the plywood was <10%. In this case, the cohesion failure of the adhesive layer was observed. The addition of 6% PAE to the SPIA increased the wet strength by 84.8% to 0.78 MPa. The cross-linking between PAE and SPI contributed primarily to the enhanced performance, which was in accordance with our previous study [47]. However, due to the small amount of added PAE, the damaged surface showed minor morphological changes, with few wood tears. The wood failure rate of the plywood was ~30%, as shown in Figure 5c (2-1 and 2-2). With the introduction of FGO (SPI/PAE/FGO-0.4), the plywood wet strength further increased to 1.18 MPa, which was 51.3% higher than that of the SPI/PAE adhesive, and 180.9% higher than that of the SPIA due to two reasons: First, the GO sheet structure provides a microscopic orientation in addition to toughness, and thereby facilitates the formation of a layered structure within the adhesive (Figure 6). Second, a denser cross-linked network structure formed in the presence of PAE increases the adhesive compactness and water resistance. The surfaces of adhesive SPI/PAE/FGO were rough with wood burrs. The plywood failure rate reached 90%. The wood failure rate increased significantly due to the formation of internal cross-links in the adhesive structure, resulting in increased cohesion and penetration of the adhesive into the wood, and the formation of a firm bond between the adhesive and the wood. When the FGO content was further increased, the wood failure rate decreased, due to the introduction of excessive amino groups, which reduced the adhesive’s water resistance. Meanwhile, the viscosity of the adhesive was increased dramatically, which interfered with the coating process. The aggregation of large black adhesive is visible on the board’s surface (Figure 5c: 5-2). The uneven coating reduced the bonding strength and wood failure rate. The changes in the tensile failure and surface roughness of the plywood were consistent with the altered bonding strength. Further, the construction of a hyper-branched and organic–inorganic hybrid structure greatly reduced the cross-linking agent concentration up to 78.6% compared with traditional adhesives containing PAE, resulting in cleaner and more sustainable adhesives (Figure 5b).

#### 3.2.4. Surface Cracks and Cross-Sectional Morphology

A tough adhesive was required for the plywood to balance the interior force in the panel, which improved the bonding performance. The adhesive toughness was determined by its surface cracks (Figure 6). The presence of numerous small holes and cracks on the SPI adhesive suggested brittleness. However, when the PAE was added, the pores disappeared, but additional and larger cracks appeared, indicating that the PAE improved the brittleness. Introduction of a small amount of FGO into the adhesive system reduced the number of cracks. As the amount of FGO increased, the cracks almost completely disappeared. These results indicate that the adhesive increased in toughness in response to the layered structure of the FGO. 

**Figure 7:**
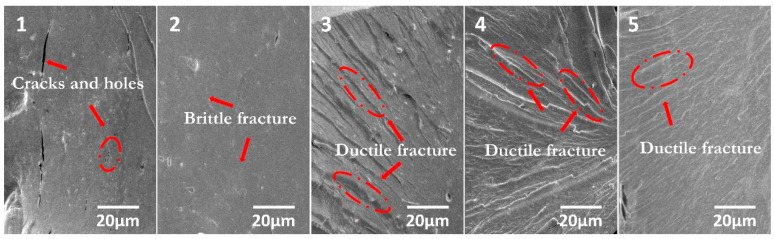
SEM images of fracture surfaces of cured adhesives: 1: SPI adhesives, 2: SPI/PAE adhesives, 3: SPI/PAE/0.2% FGO adhesives, 4: SPI/PAE/0.4% FGO adhesives, and 5: SPI/PAE/0.6% FGO adhesives.

The cross-sectional morphology of different adhesives is shown in Figure 7. Notably, numerous cracks appeared in the pure SPI adhesive cross-section because of the solidification of the non-cross-linked proteins at high temperatures, and the destruction of weak bases combining with proteins during water evaporation [12]. The presence of additional pores and cracks leads to water diffusion through the adhesive, resulting in bond failure. In contrast, the pores and cracks disappeared from the SPI/PAE adhesive surface, which is attributed to the formation of cross-linked structures. Additionally, both presented a smooth, flat fracture surface with significant brittleness, indicating that curing resulted in increased brittleness of the two adhesives. These results are consistent with those obtained from macroscopic slide analysis. The SPI/PAE/FGO samples were compact and had many pleat tears, thereby demonstrating a ductile fracture. The compactness resulted from the three-dimensional network structure formed by the cross-linking reaction, and the formation of wrinkles can be attributed to the toughening induced by the FGO layered structure.

## 4. Conclusions

In this work, HBPA was successfully grafted onto GO via reaction between the epoxy group and the N-H in HBPA, which were further combined with SPI and PAE to form an organic–inorganic hybrid structure inspired by nacre and naturally occurring branched structures. The results showed that when only 0.4 wt% FGO and 0.75 wt% PAE were added to the SPIA formulation, the water resistance of the adhesive improved by 66.5% using the sol-gel method. The wet shear strength of the resulting adhesive compared with the pure SPIA increased from 0.45 to 1.18 MPa, an increase of 181%, and the wood breaking rate of the plywood reached 90%. Compared with traditional chemical cross-linking modification involving PAE, the amount of cross-linking agent used in this study was reduced by nearly 78.6% and was substantially less than the levels of different cross-linkers added in other adhesives, due to the hyperbranched cross-linking between protein molecules, cross-linking agent, and FGO. In addition, the sheet structure of GO and the flexible macromolecular structure of the hyperbranched compound improved toughness and thermostability, which further enhanced the bonding performance of adhesive. This strategy provides an environmentally friendly and efficient strategy to develop biomass adhesives with high strength and toughness. The method can also be applied to develop low-cost nanomaterials or micromaterials as well as reinforced composites and polymers.

## Figures and Tables

**Table 1:** Different adhesive formulations.

Samples	Adhesive Formulations
1 SPI	SPI (8 g) + Deionized water (92 g)
2 SPI/PAE	SPI (8 g) + Deionized water (86 g) + PAE ^a^ (6 g)
3 SPI/PAE/0.2% FGO	SPI (8 g) + Deionized water (85.8 g) + PAE (6 g) + FGO (0.2 g)
4 SPI/PAE/0.4% FGO	SPI (8 g) + Deionized water (85.6 g) + PAE (6 g) + FGO (0.4 g)
5 SPI/PAE/0.6% FGO	SPI (8 g) + Deionized water (85.4 g) + PAE (6 g) + FGO (0.6 g)

a—The solid content of PAE is 12.5%.

**Table 2:** The values of temperatures at 5%, 10%, and 50% of mass loss of different adhesives.

Samples	Temperatures at 5%, 10%, and 50% Mass Loss (°C)		
	5%	10%	50%
1 SPI	111.8	247.8	338.9
2 SPI/PAE	211.8	255.8	342.3
3 SPI/PAE/0.2% FGO	212.2	261.2	353.4
4 SPI/PAE/0.4% FGO	202.4	257.3	353.2
5 SPI/PAE/0.6% FGO	172.9	248.2	347.9

## Data Availability

The authors declare no conflict of interest.
